# Neoantigens in Hematologic Malignancies

**DOI:** 10.3389/fimmu.2020.00121

**Published:** 2020-02-14

**Authors:** Melinda A. Biernacki, Marie Bleakley

**Affiliations:** ^1^Clinical Research Division, Fred Hutchinson Cancer Research Center, Seattle, WA, United States; ^2^Department of Medicine, University of Washington, Seattle, WA, United States; ^3^Department of Pediatrics, University of Washington, Seattle, WA, United States

**Keywords:** neoantigen, hematologic malignancies, human leukocyte antigen, T cell receptor, immunotherapy, mutations, fusion proteins

## Abstract

T cell cancer neoantigens are created from peptides derived from cancer-specific aberrant proteins, such as mutated and fusion proteins, presented in complex with human leukocyte antigens on the cancer cell surface. Because expression of the aberrant target protein is exclusive to malignant cells, immunotherapy directed against neoantigens should avoid “on-target, off-tumor” toxicity. The efficacy of neoantigen vaccines in melanoma and glioblastoma and of adoptive transfer of neoantigen-specific T cells in epithelial tumors indicates that neoantigens are valid therapeutic targets. Improvements in sequencing technology and innovations in antigen discovery approaches have facilitated the identification of neoantigens. In comparison to many solid tumors, hematologic malignancies have few mutations and thus fewer potential neoantigens. Despite this, neoantigens have been identified in a wide variety of hematologic malignancies. These include mutated nucleophosmin1 and PML-RARA in acute myeloid leukemia, ETV6-RUNX1 fusions and other mutated proteins in acute lymphoblastic leukemia, BCR-ABL1 fusions in chronic myeloid leukemia, driver mutations in myeloproliferative neoplasms, immunoglobulins in lymphomas, and proteins derived from patient-specific mutations in chronic lymphoid leukemias. We will review advances in the field of neoantigen discovery, describe the spectrum of identified neoantigens in hematologic malignancies, and discuss the potential of these neoantigens for clinical translation.

## Introduction

**Figure 1:**
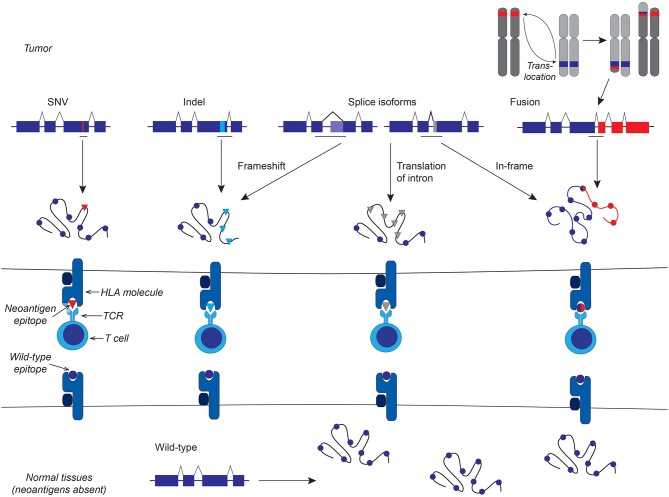
Schematic representation of different classes of non-viral neoantigens. From left to right: Protein-coding single nucleotide variants (SNV) lead to neoantigens that differ from the wild-type antigen by a single amino acid that alters HLA and/or TCR binding. Frameshift insertion-deletion (indels) mutations result in a novel amino acid sequence downstream of the indel. Cancer-specific splice isoforms can lead to frameshifts, if the splice is out of frame, or, like genomic fusions, juxtapose two usually separate amino acid sequences, or produce entirely novel amino acid sequence from introns or other portions of the genome that are not normally translated.

Neoantigens are composed of peptides derived from full-length aberrant cancer-specific proteins through a multi-step intracellular process that has been extensively reviewed (1–3) and presented in complex with human leukocyte antigen (HLA) molecules. This peptide-HLA complex is recognized by T cell receptors (TCRs). Non-viral neoantigens can be potentially be generated from any protein-coding mutations, fusion proteins, and cancer-specific splice isoforms (Figure 1), although not every aberrant protein will yield neoantigens. In the treatment of solid tumors, clinical successes have been seen with adoptive transfer of neoantigen-specific tumor-infiltrating lymphocytes (TIL) (4–7) and neoantigen vaccines (8–10), highlighting the importance of this class of antigens in effective anti-tumor immunity. T cell responses against neoantigens also appear to contribute to the efficacy of immune checkpoint blockade therapy (11, 12) and allogeneic hematopoietic cell transplantation (HCT) (13). Therapies targeting a neoantigen derived from an oncogenic driver in the founding clone could be curative, and tumor escape through loss of the target protein is unlikely when the neoantigen is from a protein critical for maintaining the malignant phenotype. As neoantigens are presented solely on malignant cells and not on healthy equivalents, the risk of “on-target, off-tumor” toxicity is minimized.

There are three major limitations to therapeutically targeting neoantigens in hematologic malignancies. First, most hematologic malignancies have relatively few protein-coding mutations and/or gene fusions, and thus fewer potential neoantigens than solid tumors, which may carry hundreds or even thousands of mutations in an individual patient (14). Second, therapies directed against any one neoantigen will apply only to the subset of patients who have both the mutation or fusion and restricting HLA allele, making neoantigens a less broadly applicable target than antigens from overexpressed wild-type proteins. However, in contrast to most solid tumors, many hematologic malignancies have recurrent mutations and/or fusions that are common within subgroups of patients and represent shared neoantigens. Finally, though targeting driver-derived neoantigens may prevent escape through loss of the target protein, other mechanism of escape from neoantigen-directed immunotherapy are possible, including downregulation or loss of HLA expression (15–24) or altered proteasomal processing of the epitope (25) by the malignant cell. However, the potential limitations of neoantigens as therapeutic targets are outweighed by their benefits: the high specificity for tumor and absent expression on normal cells; the ability to target intracellular as well as cell surface proteins; and, in some cases, the indispensable role of the aberrant protein in the malignant phenotype (26). Targeting a single high-quality neoantigen can be sufficient for disease control or even cure (4, 6, 7).

## Neoantigen Discovery

Innovations in high-throughput genomic and transcriptomic sequencing techniques have greatly facilitated the identification of protein-coding mutations and fusions that produce potential neoantigens. However, there is still no reliable comprehensive *in silico* method for identifying immunogenic neoantigen epitopes from protein-coding mutations, splice variants, or other amino acid sequence-altering abnormalities. One challenge is determining which peptides will be presented on HLA molecules (27). *In silico* HLA-binding prediction algorithms [including but not limited to (28–36)] can predict binding of peptides to HLA molecules with reasonable accuracy and thereby identify candidate neoantigen epitopes. HLA-binding prediction algorithms are quite robust for prevalent HLA class I molecules, and active research by multiple groups has led to a greater understanding of and an improved ability to reliably predict peptide binding to uncommon class I molecules and most class II molecules (37–44). However, HLA-binding predictions do not specify whether the peptides are processed and presented on cell surfaces, although separate predictive tools for antigen processing have been developed (45–53). HLA-binding prediction from the parent amino acid sequence will additionally miss non-canonical epitopes (54), such as post-translationally modified or spliced peptides (55), and will also miss epitopes that are not predicted to bind HLA but actually do (56). An alternative approach is to directly determine the peptidome of malignant cells by immunoprecipitating HLA complexes and then to elute and identify peptides by tandem liquid chromatography–mass spectrometry (57–59). This unbiased approach can identify peptides as they are naturally presented on cells of interest but has significant technical hurdles (60–63). Modifications, such as the use of monoallelic cells (43, 60) should help to overcome some of these technical issues. Since predictive algorithms rely on datasets of peptides that are naturally processed and HLA-binding, improvements to direct identification of HLA ligands will in turn increase the reliability of predictive tools (64).

Determining which epitopes are immunogenic is also a challenge. Presentation of a peptide epitope on an HLA molecule is necessary but not sufficient for T cell recognition. Currently there are no reliable *in silico* tools to assess the immunogenicity of a neoantigen peptide, although this is an active area of research (65–70). There are three starting pools of cells in which one can assess the immunogenicity of a putative neoantigen: patient TIL or marrow-infiltrating lymphocytes (MIL), patient peripheral blood T cells, and healthy donor peripheral blood T cells after primary *in vitro* stimulation. The T cell repertoire of patients may be enriched for neoantigen-specific T cells (71, 72) due to antigen-induced expansion, but immunosuppressive tumor environments can render such T cells dysfunctional (73) or even absent (74). Finally, while neoantigen-specific T cells may exist in the repertoires of patients, unbiased methods to determine the cognate antigens of TCRs from their sequence are still in their infancy (23, 75). Stimulating healthy donor T cells with neoantigen-bearing antigen-presenting cells *in vitro* can be used to isolate reactive T cells without confounding T cell dysfunction (76, 77).

## Shared vs. Personal Neoantigens

Shared or public neoantigens derive from aberrant proteins that are present in all or a sizeable subset of patients with a given disease. In contrast, private or personal neoantigens are those that result from mutations, fusions, or other abnormal amino acid sequences that occur rarely in a disease or are idiosyncratic to an individual's malignancy.

Whole genome and whole exome sequencing of hematologic malignancies (78–85) has revealed the spectrum of fusions and mutations (also referred to as the mutanome) of these diseases, including events that range from rare to highly recurrent. Many of these genetic abnormalities may give rise to neoantigens. Mutanomes provide a rich source of cancer-specific aberrant amino acid sequences that can be interrogated with HLA-binding prediction algorithms to identify candidate neoantigens (70). However, with the exception of one study in the myeloproliferative neoplasms (MPNs) (85), the mutanomes of hematologic malignancies have not yet been thoroughly explored as sources of neoantigens. Another source of both public and personal candidate neoantigens is the HLA peptidome, which is the comprehensive library of peptides eluted from HLA molecules isolated from malignant primary cells and/or cell lines and characterized by mass spectrometry. HLA peptidomes have been defined in acute myeloid leukemia (AML) (57), chronic lymphocytic leukemia (CLL) (59, 86), multiple myeloma (87), and chronic myeloid leukemia (CML) (58). However, mutation-derived candidate neoantigen epitopes have only been identified in more focused HLA peptidome studies [for example, in a subset of AML (74, 88)], reflecting both the heterogeneity of these diseases and the currently limited sensitivity of this approach.

Personal neoantigens can arise from truly patient-specific gene mutations and fusions. In addition, some recurrently affected single genes and gene fusions are highly heterogenous across individuals and would be expected to yield semi-personal rather than shared neoantigens. For example, fusions involving the mixed lineage leukemia (MLL)/ histone-lysine N-methyltransferase 2A (KMT2A) gene in acute lymphoblastic leukemia (ALL) and AML produce diverse amino acid sequences among patients because the fusions may occur at multiple breakpoints in the MLL/KMT2A genes and with multiple (>100) partner genes (89, 90). Recurrently mutated gene in hematologic malignancies that are likely to produce semi-personal rather than shared neoantigens include Wilms tumor 1 (WT1) in AML (91–94) and T cell ALL (95), Notch1 and FBXW7 in T cell ALL (96–98), and TP53 in multiple malignancies (99–101). In these examples, mutations occur at a variety of sites in the gene and involve multiple different nucleotide substitutions, insertions, and/or deletions, such that few, if any, of the resulting amino acid sequences and resulting potential neoantigens would be shared among patients even with the same disease.

At the other end of the spectrum are highly recurrent fusions and mutations, exemplified by the RUNX1-RUNX1T1 fusion (89) and exon 12 mutations in nucleophosmin1 (NPM1) (102, 103) in AML. Virtually all patients with such fusions or mutations will have identical aberrant amino acid sequences. Neoantigens created from these abnormalities are shared among patients who have the mutations or fusions and are potential therapeutic targets for these individuals as a group.

Whether optimal therapies should target shared neoantigens, personal neoantigens, or both is currently unknown. Some key features of neoantigen quality have been postulated [reviewed in (104)], including: clonality, dissimilarity to self-antigens, similarity to microbial antigens (105), high protein expression, binding to HLA, and low likelihood that genetic abnormality yielding the neoantigen will be lost (for example, driver mutations or genes involved in cell survival where loss would harm cancer fitness) through deletion or transcriptional repression. Neoantigens with high-quality features are likely to be suitable therapeutic targets whether they are personal or shared. One note of caution with personal neoantigens is that unless autologous tumor is available, there may be no way to validate that a given putative neoantigen is in fact presented on primary tumor, and thus no way to confidently predict therapeutic efficacy.

The feasibility of targeting personal neoantigens is currently under investigation. As the accessibility of whole genome and whole exome sequencing increases, defining an individual patient's mutanome is becoming increasingly practical, although the ability to reliably predict personal neoantigens remains imperfect (27). Personalized neoantigen vaccines based on patient mutanomes have shown efficacy in solid tumors (8–10), and as of December 2019, 14 clinical trials of personalized neoantigen vaccines were recruiting in the United States, although only one of these studies includes patients with a hematologic malignancy (NCT03631043, multiple myeloma). In addition, increasingly sophisticated T cell engineering technologies have made the production of personalized neoantigen-specific engineered T cell therapies more practical; currently three trials of such therapies for patients with advanced solid tumors (NCT03412877, NCT04102436, NCT03970382) are enrolling.

Although both personal and shared neoantigens have therapeutic promise, in this review we will focus primarily on shared neoantigens (summarized in Table 1), which make up the bulk of the data to date.

**Table 1:** Shared or potentially shared neoantigens relevant in hematologic malignancies (fs, frameshift).

**Disease**	**Parent protein**	**Epitope**	**HLA restriction**	**Level of evidence for neoantigen status**	**Reference(s)**
AML	NPM1 fs (type A/D)	C*LAVEESL	A*02:01	Definite	(74, 88)
		CLAVEEVSL			
		AIQDLCLAV		Possible	(106–109)
	NPM1 fs (type C)	AIQDLCVAV		Possible	
	PML-RARA	NSNHVASGAGEAAIETQSSSSEEIV	DR*11	Possible	(110, 111)
ALL	ETV6-RUNX1	RIAECILGM	A*02:01	Conflicting	(112–114)
MPN	CALR fs	KMRMRRMRR	A*03:01	Possible	(85, 109, 115)
		RMRRTRRKM	B*07:02	Possible	
		Multiple	B*08:01	Candidate	(85)
		RMMRTKMRM	C*03:03	Possible	(109)
	JAK2 V617F	VLNYGVCFC	A*02:01	Possible	(116)
	MPL	Multiple	A*03:01	Candidate	(85, 116)
CML	BCR-ABL1	KQSSKALQR	A*03:01	Possible	(117)
	BCR-ABL1 E255K	KVYEGVWKK	A*03:01	Possible	(118)
B cell lymphomas	D393-CD20	(P)LFRRMSSLEVIA	DRB1*04	Possible	(119)
Multiple tumors	KRAS G12D	GADGVGKSA(L)	C*08:02	Definite	(6)
		VVVGADGVGK	A*11:01	Possible	(120)
	KRAS G12V	(V)VVGAVGVGK		Possible	
	BRAF V600E	GDFGLATEKSRWSGS	DQA1*03/DQB1*03	Possible	(71)
	TP53 R175H	HMTEVVRHC	A*02:01	Possible	(121, 122)
		YKQSQHMTEVVRHCPHHERCSDSDG	Class II	Possible	(121)
	TP53 R248Q	YMCNSSCMGGMNQRPILTIITLEDS	Class I & II	Possible	
	TP53 R248W	SSCMGGMNWR	A*68:01	Possible	
		SSCMGGMNWRPILTII	DPB1*02:01	Possible	
	TP53 R282W	FEVRVCACPGRDWRTEEENLRKKGE	Classs II	Possible	

*For levels of evidence*:

*“Definite” indicates that the epitope is immunogenic and that epitope-specific T cells clearly and consistently recognize primary malignant cells in a mutation/fusion- and HLA-specific manner*.

*“Possible” indicates epitopes that have demonstrated immunogenicity but either lack direct evidence of specific recognition of primary malignant cells (i.e., cell lines only) or data is inconsistent*.

*“Candidate” indicates that the peptide epitope has been demonstrated to bind the restricting HLA in vitro*.

*“Controversial” indicates conflicting data between groups*.

## Neoantigens in Specific Hematologic Malignancies

### Acute Myeloid Leukemia

AML is the most common acute leukemia in adults, and mutations in nucleophosmin1 (NPM1) occur in 30–35% of adult patients (102, 103). The majority of NPM1 mutations are insertions of four nucleotides in exon 12, resulting in a frameshift that produces a novel C-terminal 11 amino acid sequence (123). NPM1 mutations are stable across the disease course and considered to be driver events, thus an optimal immunotherapy target. Eighty-five percent of patients with NPM1-mutated (NPM1^mut^) AML share the type A/D mutations that produce an identical abnormal amino acid sequence. Epitopes from the mutated region were independently identified as HLA ligands by two groups that used mass spectrometry to determine the amino acid sequences of peptides eluted off HLA molecules from primary leukemic blasts (74, 88) or AML cell lines (88).

Van der Lee et al. subsequently identified CD8^+^ T cell clones from healthy donors that were specific for the NPM1^mut^ HLA-A*02:01-restricted epitopes CLAVEEVSL and C*LAVEEVSL (74). These clones specifically recognized HLA-A*02:01^+^ peptide-pulsed targets and NPM1^mut^ AML blasts. One C*LAVEEVSL-specific TCR was sequenced and transferred into CD8^+^ T cells using a viral vector. T cells with transferred NPM1^mut^ TCRs could lyse NPM1^mut^ but not NPM1 wild-type AML *in vitro* and partially controlled leukemia *in vivo* in an NPM1^mut^ OCI-AML3 cell-line-derived xenograft murine model. These results convincingly demonstrate that CLAVEEVSL and C*LAVEEVSL are naturally presented on HLA-A*02:01 on leukemic blasts, are immunogenic, and are thus *bona fide* AML neoantigens. Curiously, although the peptide was immunogenic, the authors were unable to identify naturally occurring epitope-specific T cell responses in HLA-A*02:01^+^ patients with NPM1^mut^ AML. While a subsequent publication (123) suggested that NPM1^mut^ -specific responses could be elicited *ex vivo* in patients, these studies were less stringently controlled.

Earlier studies identified candidate NPM1^mut^-derived epitopes predicted to bind HLA-A*02:01 (106–108), against which the authors elicited CD8^+^ T cells responses in patients and healthy donors after *ex vivo* stimulation. CD8^+^ T cells specific for two epitopes (AIQDLCLAV and AIQDLCVAV) identified in these publications lysed an NPM1^mut^ AML sample (106), suggesting that these epitopes were naturally processed and presented. However, these peptides were not identified among HLA-A*02:01 ligands in either of two subsequent studies that directly examined peptide epitopes eluted from HLA-A*02:01 on NPM1^mut^ primary blasts (74, 88) or cell lines (88), and identification of AIQDLCL/VAV-specific CD8^+^ T cells has not been reproduced by other groups.

In around 13% of AML cases (124, 125), a fusion of the retinoic acid receptor (RARA) gene on chromosome 17 and the promyelocytic leukemia (PML) gene on chromosome 15 occurs as a result of the chromosomal translocation, t(15; 17)(q24.1;q21.1), the classic translocation that produces the distinct entity of acute promyelocytic leukemia (APL). The resulting PML-RARA fusion protein not only serves as a driver of the leukemic phenotype but also represents a potential shared neoantigen at the fusion junction. Gambacorti-Passerini et al. investigated the immunogenicity of the PML-RARA fusion region by stimulating peripheral blood mononuclear cells (PBMC) from healthy volunteer donors with a 25mer peptide spanning the fusion (110, 126). CD4^+^ T cell clones from one donor proliferated specifically in response to exogenous PML-RARA peptide presented on HLA-DR*11 in autologous target cells. One T cell clone could lyse peptide-pulsed autologous target cells and recognize autologous target cells transduced to express the PML-RARA fusion protein. However, in a subsequent study, no PML-RARA-specific CD4^+^ T cell responses could be elicited from any of four HLA-DR*11^+^ individuals in remission after treatment for APL (111). Since neither study evaluated whether PML-RARA-specific T cells could recognize primary APL cells, the PML-RARA/HLA-DR*11 epitope would still currently be considered a possible, rather than definite, AML neoantigen pending confirmation that the epitope is naturally presented on APL cells.

### Acute Lymphoblastic Leukemia

ALL is the most common childhood cancer. Like other hematologic and pediatric malignancies, there are few non-synonymous mutations (14, 127) and thus few potential neoantigens. However, in recent studies, Zamora et al. found surprisingly abundant neoantigen-specific CD8^+^ T cell responses in MIL from pediatric patients with ALL (112). To identify putative patient-specific neoantigens, cancer-specific mutations were identified from genomic sequencing of diagnostic biopsies and matched germline tissues from six patients. HLA typing was extrapolated from sample mRNA sequencing data, and the amino acid sequences of protein-coding mutations were interrogated using HLA-binding prediction algorithms. Mutation- or fusion-derived 15mer synthetic peptides were used to evaluate patient T cell specificity *ex vivo*. Functional CD8^+^ T cell responses against at least one neoantigen were detected in all patients and encompassed 31 of 36 putative neoantigens mostly originating from patient-specific single gene mutations.

The Zamora study also identified T cells responsive to several epitopes from the recurrent ETV6-RUNX1 fusion in five patients. The ETV6-RUNX1 fusion results from the t(12; 21)(p13.2;q22.1) chromosomal translocation and is the most common genetic event in childhood B-lineage ALL, occurring in 15–20% of patients (128–131). ETV6-RUNX1 epitopes eliciting T cell responses in this study were predicted to bind to HLA-A*02:01, HLA-A*11:01, and HLA-B*15:01, and ETV6-RUNX1-specific T cells were identified by positive staining with HLA-A*02:01 or HLA-A*11:01 peptide/HLA tetramers. In earlier studies, the same HLA-A*02:01 epitope (RIAECILGM) was identified as binding stably to HLA-A2 in *in vitro* competitive-binding assays by a group that also isolated CD8^+^ T cell lines specific for the epitope (113). The two RIAECILGM-specific lines that were isolated from healthy donors lysed fusion-expressing cell lines, and one T cell line from a patient with ETV6-RUNX1^+^ ALL lysed autologous leukemic blasts at low levels. However, a subsequent study disputed whether the ETV6-RUNX1 epitope is in fact naturally processed and presented, as it showed that the native RIAECILGM peptide had virtually no binding to HLA-A*02:01 *in vitro*, was not processed by cells transduced to express the ETV6-RUNX1 epitope, and was not cleaved at the relevant position by human proteasomes *in vitro* (114). Given the conflicting data, it remains unclear whether the RIAECILGM epitope is truly an ALL neoantigen.

### Myeloproliferative Neoplasms

Philadelphia (Ph) chromosome-negative myeloproliferative neoplasms (MPNs) comprise a group of disorders, including essential thrombocytosis (ET), polycythemia vera (PV), and primary myelofibrosis (PMF). MPNs arise from an abnormal hematopoietic progenitor cell, in most cases consequent to the acquisition of one of three driver mutations in JAK2 (Janus kinase 2), CALR (calreticulin), or MPL (c-mpl proto-oncogene; thrombopoietin receptor), along with a variety of passenger mutations (132) that can all produce neoantigens. Recently, Schischlik et al. comprehensively evaluated potential neoantigens in 113 patients with MPNs (85). Using whole-transcriptome sequencing to define the MPN mutanome, they identified 13 fusions, 221 non-synonymous single nucleotide variants, 31 insertion or deletion mutations, and 20 frameshift-producing splicing abnormalities. HLA-binding predictions for the 12 most prevalent HLA-A, -B, and -C alleles in their patient cohort yielded 541 patient-specific peptides predicted to bind to at least one of the HLA alleles. Subsequent *in vitro* HLA binding studies of 35 peptides derived from aberrantly spliced proteins associated with SF3B1 mutations and from mutated CALR (CALR^mut^) and MPL validated binding of 23 peptides to HLA-A*03:01, -A*11:01, -B*07:02, and -B*08:01.

Although Schischlik et al. did not evaluate processing or immunogenicity of their putative neoantigens, others have identified T cell responses to CALR^mut^ and JAK2 V617F. Cimen Bozkus et al. used *in vitro* stimulation to elicit T cell responses to CALR^mut^ peptides that were primarily CD4^+^ T cells in patients with MPNs and both CD4^+^ and CD8^+^ in healthy donors. Inhibition of the PD-1 and CTLA-4 immune checkpoint molecules *in vitro*, and PD-1 *in vivo* (in a patient treated with pembrolizumab), enhanced T cell responses. An immunogenic HLA-C*03:03-restricted 10mer epitope was identified, and T cells specific for this epitope produced cytokine in response to antigen-presenting cells pulsed with a 15mer peptide, indicating that the epitope was processed from the longer peptide (109). While this finding is encouraging, data conclusively demonstrating that the CALR^mut^ epitope is processed from the full-length protein and presented on HLA-C*03:03 on primary MPN cells is currently lacking. Another group described cytokine production, primarily by CD4^+^ T cells, in response to *ex vivo* stimulation of peripheral blood mononuclear cells from patients with MPNs with long (31mer) CALR^mut^ peptides (133). CD8^+^ T cells specific for CALR^mut^ peptides presented on HLA-A*03:01 and -B*07:02 were identified by another group, but the low avidity of the T cells prevented them from assessing whether the epitopes were naturally processed and presented on CALR^mut^ cells (115). Additionally, a 9mer peptide spanning the JAK2 V617F mutation (VLNYGVCFC) was identified as a ligand of HLA-A*02:01 by HLA-binding prediction; epitope-specific CD8^+^ T cells lysed target cells either pulsed with the mutant peptide or naturally expressing JAK2 V617F, but also recognized targets pulsed with wild-type JAK2 peptide with lower efficiency (116). While this is a promising possible neoantigen with broad applicability for patients with MPNs, especially PV, further study is needed to definitively show that VLNYGVCFC is presented on primary malignant cells.

### Philadelphia Chromosome-Positive Malignancies

The BCR-ABL1 fusion derives from translocation t(9; 22)(q34;q11), also called the Ph chromosome, which is highly recurrent in chronic myeloid leukemia (CML) and Ph-positive ALL (Ph^+^-ALL). Most patients have one of two fusions resulting from different breakpoints, namely ^p210^BCR-ABL1 and ^p190^BCR-ABL1. ^p210^BCR-ABL1 is found in both CML and Ph^+^-ALL, while ^p190^BCR-ABL1 is primarily associated with Ph^+^-ALL (134). As an oncogenic driver, the BCR-ABL1 fusion is essential to the malignant phenotype and an ideal therapeutic target. Small molecule tyrosine kinase inhibitors (TKIs) are now key components of therapy for Ph^+^ malignancies, but resistance does occur. Because the fusion is highly recurrent and disease-specific, it is a potential source for shared neoantigens. BCR-ABL1 was first described as a neoantigen in 1992 (135), and additional BCR-ABL1 epitopes were subsequently investigated by multiple groups [reviewed in (136)]. However, evidence for the natural CML presentation of BCR-ABL1 fusion peptides is conflicting: one group eluted an immunogenic fusion peptide from HLA-A*03:01 in primary CML (117), but a recent comprehensive evaluation of the HLA-ligandome in CML found no BCR-ABL1 epitopes presented on class I or class II molecules (58). Because the biology and specific fusions differ in the two diseases, the CML and Ph^+^-ALL peptidomes may differ. Interestingly, adoptive transfer of *ex vivo*-expanded ^p190^BCR-ABL1-specific CD8^+^ T cells showed encouraging anti-leukemic activity in three patients with Ph^+^-ALL (137). Specific BCR-ABL1 mutations that confer TKI resistance might also serve as neoantigens; one group identified donor-derived CD8^+^ T cell responses to an HLA-A*03:01-restricted epitope from BCR-ABL1 E255K in a patient with the mutation who had achieved remission after HCT (118). While the BCR-ABL1 E255K-specific T cell clones could recognize minigene-transduced target cells, recognition of primary CML was not tested and thus it remains unclear whether the epitope represents a *bona fide* CML neoantigen.

### Lymphomas and Chronic Lymphocytic Leukemia

In B cell malignancies, such as lymphomas and myelomas, neoplastic B cell-produced clonal immunoglobulin (Ig) was first described as a tumor-specific antigen in 1972 (138). Ig idiotypes have been extensively investigated as neoantigens with varying degrees of success, including in clinical trials (139). More recently, Khodadoust et al. recovered peptides representing somatic mutations in Ig heavy and light chain genes from the peptidomes of both class I and class II molecules in 17 primary mantle cell lymphomas (MCL) and two MCL cell lines, and detected circulating functional CD4^+^ T cells specific for one Ig neoantigen that could kill autologous lymphoma (140). Subsequent studies by this group identified primarily class II-restricted Ig-derived neoantigens in other B cell malignancies, including follicular lymphoma, diffuse large B cell lymphoma, and chronic lymphocytic leukemia (CLL) (141). A cytoplasmic variant of CD20 (D393-CD20), produced by alternative splicing of the CD20 transcript, is detectable in malignant primary B cells and B cell lines, but not normal resting B cells (142). CD4^+^ T cell responses to an epitope of D393-CD20 could be elicited from both healthy donors and patients with B cell lymphomas after *in vitro* peptide stimulation and blocked with anti-HLA-DR monoclonal antibody, but the exact HLA restriction could not be determined (119). *MYD88* is recurrently mutated in a variety of B cell malignancies and has been proposed as a potential neoantigen (143). Separately, in a small cohort of CLL patients evaluated after HCT, CD8^+^ responses to neoantigens created from patient-specific non-Ig somatic mutations were identified; one well-studied patient-derived T cell clone could lyse autologous primary CLL cells, indicating that the epitope the clone recognized was a true personal neoantigen (13).

### Neoantigens With General Applicability in Hematologic Malignancies

While some genetic abnormalities are specific to or even defining of particular cancer types, others, especially mutations in oncogenes or tumor suppressor genes, can be found in numerous cancers with a wide variety of cellular origins, including hematopoietic tissues. For example, somatic mutations affecting members of the Ras-MAPK pathway are among the most common in human cancers and are found across diverse cancer types (144–147). Similarly, TP53 is the most commonly mutated gene in human cancer, with TP53 mutations estimated to occur in ~25% of all cancers (99). Neoantigens derived from these mutations may thus be shared not just among patients with a single disease but across patients with many different cancers, including hematologic cancers (Table 2).

**Table 2:** Recurrently mutated genes in cancers, including hematologic malignancies, for which possible or definite public neoantigens have been identified.

**Gene/gene family**	**Overall prevalence of any mutation in the gene/gene family in human cancers**	**Prevalence of any mutation in the gene/gene family in hematologic malignancies**	**Mutation hotspots (all cancers including hematologic)**	Mutations yielding possible/definite neoantigens^*^	**References**
KRAS/NRAS/HRAS	~25% (all RAS genes)	~26% multiple myeloma	G12, G13, Q61	G12D, G12V	(146, 148, 149).
		~16% AML			
		~14% ALL			
		~10% CLL			
		~5% MDS (~30% CMML)			
BRAF	~8%	~100% hairy cell leukemia	V600	V600E	(148, 150–154)
		~40–60% systemic histiocytoses			
		~5% CLL			
TP53	~25%	14% AL	R175, R245, R248, R273, R282	R175H, R248Q, R248W, R282W	(99–101, 155)
		12% AML			
		7–10% CLL			
		6% MDS			
		6–24% B cell lymphoma			
		7–40% non-B cell lymphoma			
		6% myeloma and other plasma cell dyscrasias			

* *See Table 1 for specific details about neoantigens*.

Mutations in KRAS or NRAS are found in ~5–26% of hematologic malignancies (146, 148) (Table 2). The most recurrent oncogenic mutations that occur in the RAS genes (NRAS, KRAS, HRAS) across cancers occur at codons 12, 13, and 61. As such, neoantigens derived from these recurrent mutations in RAS genes are attractive therapeutic targets with applicability in multiple diseases, including blood cancers. Moreover, the amino acid sequences of RAS family members are highly similar, such that identical epitopes may be derived from different proteins. Although there are no publications specifically investigating RAS-derived neoantigens in hematologic malignancies, findings from studies in solid tumors have potential applicability. For example, Tran et al. studied tumor-infiltrating lymphocytes (TIL) from a patient with metastatic KRAS G12D-mutated colorectal cancer and identified CD8^+^ T cell clones specific for a KRAS G12D epitope presented on HLA-C*08:02 (6). KRAS G12D specific T cells expanded in the patient's peripheral blood after re-infusion of TIL, were persistently detectable ~9 months after TIL infusion, and mediated at least a transient regression of metastatic lung lesions. Subsequently, Cafri et al. performed *in vitro* stimulation of memory T cells isolated from two patients with KRAS-mutated solid tumors (one with endometrial cancer, one with rectal cancer) and identified CD8^+^ T cells specific for an HLA-A*11:01-restricted epitope from KRAS G12V and CD4^+^ T cells specific for an HLA-DRB1*08:01 restricted epitope from KRAS G12D (156). Earlier studies also identified murine TCRs with specificity for HLA-A*11:01-restricted epitopes from KRAS G12D and G12V in HLA-A*11:01^+^ transgenic mice immunized with KRAS peptides (120). Retroviral transfer of the KRAS-specific TCRs into human T cells conferred KRAS neoantigen-specific anti-tumor activity *in vitro* and *in vivo*. These findings have been translated into clinical trials of transgenic TCR T cell immunotherapy for HLA-A*11:01^+^ patients with certain KRAS G12D- or G12V-mutated solid tumors (NCT03190941 and NCT03745326). Although this clinical trial is directed toward patients with solid tumors, such therapies also have applicability to those with hematologic malignancies; for example, alterations at codon G12 of NRAS occur in a subset of patients with AML and produce identical amino acid sequences to the equivalent KRAS mutations, and so should yield the same epitope that could be targeted with KRAS G12D or G12V-directed T cells.

Mutations in BRAF, another component of the Ras-MAPK pathway, are present in about 8% of all human cancers (150). While the majority of BRAF-mutated malignancies are solid tumors, BRAF mutations do occur in a subset of hematologic malignancies. The BRAF V600E mutation is highly prevalent in hairy cell leukemia (151–153) and systemic histiocytoses (Erdheim-Chester disease and Langerhans cell histiocytosis) (154) and have also been identified in CLL (148) (Table 2). BRAF-derived neoantigens, particularly those originating from the V600E mutation, thus have applicability in a subset of hematologic malignancies. By examining peripheral blood lymphocytes from a patient with BRAF V600E^+^ melanoma who had a clinical response after TIL therapy, Veatch et al. identified a CD4^+^ T cell clone specific for an HLA-DQB1*03-restricted epitope of BRAF V600E (71). Lentiviral transfer of the BRAF V600E-specific TCR to donor CD4^+^ conferred recognition of BRAF V600E-expressing target cells. An earlier study also detected CD4^+^ T cell responses to HLA class II-restricted epitopes from BRAF V600E in patients with BRAF V600E melanoma, although not in the context of clinical response after immunotherapy (157).

TP53 mutations occur in malignancies of all origins (99), including all types of hematologic malignancies (100, 101, 158) (Table 2). While TP53 mutations can be quite heterogeneous, there are mutation hotspots at R175, R245, R248, R273, and R282 that are shared across multiple kinds of cancers, including hematologic cancers (99, 101). Neoantigens created from TP53 mutations thus have broad potential applicability in blood cancers as well as solid tumors. Malekzadeh et al. isolated T cells specific for HLA class I- and class II-restricted epitopes from five different recurrent TP53 mutations from TIL generated from patients with a variety of epithelial tumors (colorectal, ovarian, and pancreatic) (121). Both this publication and a subsequent report from the same group (122) identified an HLA-A*02:01-restricted epitope from TP53 R175H that appears to be naturally presented on a number of tumor cell lines. CD4^+^ and CD8^+^ responses to epitopes from patient-specific TP53 mutations have also been identified (122, 159, 160). Because the codon distribution of TP53 mutations is not specific to the tissue origin of a cancer, therapy targeting the TP53 R175H epitope, for example, should be equally applicable in a TP53 R175H^+^ hematologic cancer as in a TP53 R175H^+^ solid tumor, assuming the epitope is processed and presented appropriately. Similarly, specific mutations that are identical in many different malignancies, like the ones described in this section, are sources for neoantigens that are shared across cancers.

## Therapeutic Applications of Neoantigens

While neoantigens are attractive targets for therapy because of their high specificity for malignant cells, there are challenges in translating neoantigen-directed immunotherapies to the clinic and the best approach to neoantigen-directed therapy is currently unknown. One strategy is to adoptively transfer neoantigen-specific T cells. T cells can be isolated from patient peripheral blood, TIL, or MIL (in hematologic malignancies), then expanded *ex vivo* non-specifically or against a defined antigen and re-infused (4, 6, 7, 161). Alternatively, T cells can be engineered to express a transgenic neoantigen-specific TCR (TCR-T), allowing infusion of a rapidly generated product with defined specificity and composition. Preclinical studies have shown that transfer of neoantigen-specific TCR-Ts is feasible (71, 74, 122), and two clinical trials of autologous TCR-T targeting HLA-A*11:01-restricted epitopes derived from point mutations in KRAS are enrolling (NCT03190941 and NCT03745326). Although TCR-T targeting epitopes from wild-type WT1 have shown safety (162–164) and efficacy (165), no clinical trials of neoantigen-specific TCR-T immunotherapy for hematologic malignancies have opened to date. TCR constructs can be modified to include other features to improve TCR-T safety and function: a CD8 co-stimulatory receptor enables CD4^+^ T cells to function with a class I-restricted TCR and provide targeted help to neoantigen-specific CD8^+^ T cells (166–168), a safety switch (167, 169) enables rapid removal of transgenic TCR-T cells in the event of toxicity, and other elements have been advanced [reviewed in (170)]. Lastly, vaccines do not require adoptive cell transfer, have shown clinical efficacy in solid tumors (8–10), and are particularly attractive for targeting highly immunogenic but less prevalent neoantigens.

Many factors influence which immunotherapy strategy is optimal for a given antigen. For neoantigens, the relatively low prevalence of each neoantigen among individuals with a given hematologic malignancy is a significant consideration, as immunotherapy for one neoantigen will apply only to a subset of patients. For example, the NPM1^mut^ epitope described above (74) is only presented by the ~15% of AML patients with NPM1^mut^ (30–35%) and HLA-A*02:01 (~50% in the U.S.A.) and this represents one of the most broadly applicable recurrent neoantigens in hematologic malignancies. Producing neoantigen-directed TCR-T therapies using currently standard viral transfer methods is probably not cost-effective for less common neoantigens given their narrow applicability, but vaccines could be. Moreover, the growing use of non-viral techniques for TCR gene transfer, such as transposon-based technologies (159, 171, 172), nanoparticles (173), and RNA electroporation (174) should facilitate the development of TCR-T immunotherapy for all neoantigens, as illustrated by a recently opened clinical trial of gene-edited TCR-T immunotherapy for personal neoantigens (NCT03970382). The use of “universal donor cells” that have been engineered to be HLA-negative and express natural killer (NK) cell inhibitory molecules (175), in combination with silencing or editing the endogenous TCR (163), could also facilitate neoantigen-directed TCR-T immunotherapy.

Another consideration is the natural immunologic landscape of a particular malignancy. *Ex vivo* expansion and vaccination rely largely on the presence of existing anti-tumor responses that can be boosted *in vivo* or *ex vivo* and would be challenging in an immunosuppressive environment. Because hematologic malignancies have multiple mechanisms for blocking effective naturally occurring anti-leukemic immune responses (11, 176–183), TCR-T immunotherapy may be preferable for these diseases. For example, transgenic TCRs can used to modify selected virus-specific memory T cells for therapeutic transfer (165). Immune checkpoint blockade has been used alone in MPNs (109); combining them with neoantigen-specific immunotherapies could potentiate their effect in these and other hematological malignancies.

## Discussion

Much progress has been made in the field of neoantigens generally and in hematologic malignancies specifically. A number of promising *bona fide* and potential shared neoantigens have been identified for hematologic malignancies, most of which are derived from well-established mutations and fusions. However, growing access to comprehensive sequencing technologies has greatly enhanced the ability to define disease- and patient-specific mutanomes, which are valuable sources of potential neoantigens. Combined with improvements in T cell antigen discovery approaches, sequencing advances will facilitate the discovery of additional shared and personal neoantigens derived from known as well as new genetic abnormalities, expanding the repertoire of potential targets and moving the field forward. While there are a number of challenges in translating neoantigen-directed immunotherapies to the clinic, the rapid evolution of neoantigen discovery methods and the immunotherapy field is making barriers to clinical translation surmountable. Experience gained from T cell immunotherapy and vaccine studies in solid tumors and from cell therapy engineering for non-neoantigen targets will provide a critical foundation for building potent neoantigen-directed immunotherapies that are viable treatment strategies for hematologic malignancies.

## Author Contributions

MAB and MB reviewed the literature and wrote and edited the manuscript.

### Conflict of Interest

MB has received compensation from Miltenyi Biotec for presentations at conferences and corporate symposia pertaining to research unrelated to that presented in the current manuscript. MB is a Founder and Scientific Advisory Board member of HighPassBio and Scientific Advisory Board member of Orca Bio. The remaining author declares that the research was conducted in the absence of any commercial or financial relationships that could be construed as a potential conflict of interest.
